# Deep brain stimulation improves symptoms across all dimensions in treatment-resistant depression

**DOI:** 10.1016/j.neurot.2025.e00623

**Published:** 2025-06-10

**Authors:** N. Runia, G.J.J. Mol, D.A.J.P. Denys, H. Ardon, G. Beute, M. Bot, D.A. de Waardt, D.W.W. de Knijff, R.J.T. Mocking, P. Notten, G.J.M. Rutten, P.R. Schuurman, P. van den Munckhof, J. van Laarhoven, G.A. van Wingen, I.O. Bergfeld

**Affiliations:** a Amsterdam UMC Location University of Amsterdam, Department of Psychiatry, Meibergdreef 9, Amsterdam, the Netherlands; b Amsterdam Neuroscience, Amsterdam, the Netherlands; c Department of Neurosurgery, ETZ, Location Elisabeth, Tilburg, the Netherlands; d Amsterdam UMC Location University of Amsterdam, Department of Neurosurgery, Amsterdam, the Netherlands; e Department of Psychiatry, ETZ, Location Elisabeth, Tilburg, the Netherlands

**Keywords:** Deep brain stimulation, Treatment-resistant depression, Symptom dimensions

## Abstract

Deep brain stimulation (DBS) reduces depressive symptom scores in many patients with treatment-resistant depression (TRD). However, it is unclear whether the observed improvement is similar across various symptom dimensions (e.g., anhedonia, anxiety, insomnia) or if some require additional clinical attention. Using a retrospective chart review, we assessed the trajectory of HAM-D-17 and MADRS scores during vALIC or slMFB DBS treatment within different symptom dimensions (HAM-D-17: 1) affective/anhedonia, 2) somatic/anxiety, 3) insomnia; MADRS: 1) affective/anhedonia, 2) anxiety/vegetative, 3) hopelessness) after at least a 25 ​% symptom reduction (partial response) at any time during their treatment course (n ​= ​34 for HAM-D-17, n ​= ​25 for MADRS). Results showed that each of the assessed symptom dimensions was significantly reduced compared to baseline at each of the assessed time periods (last follow-up: 2–15 years) after (partial) DBS response onset, which occurred at a median of approximately 2.5 months. Additionally, there was a significant interaction effect between symptom dimension and time period (HAM-D-17: F (12,1655.46) ​= ​5.46, p ​< ​0.001; MADRS: F (12,938.73) ​= ​2.40, p ​< ​0.01). Model coefficients indicated that insomnia symptoms (HAM-D-17) and anxiety/vegetative symptoms (MADRS) improved at a slower rate than the other symptom dimensions. Additionally, higher baseline scores in the HAM-D-17 somatic/anxiety dimension were significantly associated with a larger percentage reduction in overall symptoms after DBS (n ​= ​39, F (1,32) ​= ​12.371, p ​< ​0.01). Our findings demonstrate that DBS for TRD effectively treats depressive symptoms in all dimensions, although insomnia symptoms may improve at a slower rate, and that patients with more anxiety symptoms, who typically tend to have worse pharmacological treatment outcomes, may particularly benefit from DBS.

## Introduction

Deep brain stimulation (DBS) has been studied as an intervention for treatment-resistant major depressive disorder (MDD) for nearly 20 years [1]. Open-label clinical trials across different brain targets, such as the subcallosal cingulate gyrus (SCG), ventral anterior limb of the internal capsule (vALIC), and superolateral branch of the medial forebrain bundle (slMFB), show an average response rate (≥50 ​% symptom reduction) of approximately 50 ​% [2]. Additionally, single and double blind randomized controlled trials as well as cases with accidental stimulation discontinuation provide growing evidence that the long-term antidepressant effects of DBS cannot be attributed to chronic placebo effects [2,3].

However, although the exact rate is unclear, many TRD patients who are treated with DBS have residual depressive symptoms. For example, our group has previously shown that out of 25 patients who participated in our vALIC DBS clinical trial, 6/10 responders were clear improvers (50 ​%–75 ​% symptom reduction) and 6/15 non-responders were partial improvers (25 ​%–50 ​% symptom reduction) [4]. This suggests that DBS often relieves some but not all depressive symptoms. Furthermore, as an anatomically targeted neuromodulatory intervention, DBS is more likely to modulate symptom domains specifically associated with its neural targets, in contrast to broader-acting antidepressant treatments. Unfortunately, general practice is to report only an overall summation of all symptoms, preventing a more specific evaluation of treatment effects. Identifying symptom dimensions that exhibit suboptimal response to DBS could highlight areas requiring additional therapeutic interventions.

Depressive symptom severity is usually quantified using the Hamilton Depression Rating Scale (HAM-D) and/or the Montgomery-Åsberg Depression Rating Scale (MADRS). Both scales contain items which assess different symptoms of MDD, such as depressed mood, loss of interest, weight loss, insomnia, psychomotor agitation or retardation, fatigue, excessive guilt, difficulties concentrating, and suicidal ideations (5th ed.; DSM–5; American Psychiatric Association, 2013). A large study has shown that depressive symptom dimensions respond differently to treatment within and across different antidepressant medications [5]. For DBS, evidence for this is lacking. One single study, encompassing 20 patients with treatment-resistant depression (TRD), showed that on average, all symptom dimensions (mood, anxiety, sleep, and somatic) were significantly reduced after 1, 3, 6, and 12 months of SCG DBS compared to baseline, but with maximum reductions at different time-points for different symptom dimensions [6]. This suggests that although DBS may reduce symptoms in all dimensions, symptom dimension trajectories may be different. However, these analyses included all participating patients regardless of whether or not they showed a clinically relevant response during the treatment course. Furthermore, the onset of a clinically significant response can vary greatly among patients, due to varying durations required for optimizing DBS settings. Not taking these factors into account may have potentially distorted the results. Consequently, the effect of DBS on different symptom dimensions and their trajectories during response remains unclear. Furthermore, it is unknown whether baseline symptom severity within specific symptom dimensions is associated with DBS (non-)response.

Here, we conducted a retrospective chart review of TRD patients treated with vALIC or slMFB DBS between 2010 and 2023 ​at the Amsterdam UMC in Amsterdam and the Elisabeth-Tweesteden Hospital in Tilburg, The Netherlands. We assessed the trajectory of HAM-D and MADRS scores during (partial) response to DBS across different symptom dimensions identified through previous conducted exploratory factor analyses [7,8]. To control for the variable amount of time required to optimize DBS settings for each patient, we analyzed symptom trajectories starting from the point of partial response onset (at least a 25 ​% reduction in overall symptoms) within each patient. In addition, we assessed whether baseline symptom severity within these symptom dimensions is associated with DBS response.

## Materials and Methods

### Participants

This study concerns a retrospective chart review which did not require formal METC approval. All included patients provided written informed consent for use of their data.

Patients were included if 1) they were being treated or had previously been treated with DBS for TRD at the Amsterdam UMC in Amsterdam or Elisabeth-Tweesteden Ziekenhuis in Tilburg and 2) they provided written informed consent for the retrospective chart review. There were no exclusion criteria.

These criteria led to a sample of patients who were (being) treated with DBS as part of a previously reported clinical trial for vALIC DBS in TRD [9] (trial registration number: NTR2118), or an ongoing slMFB DBS in TRD clinical trial (trial registration number: NL8211). Generally, to be included in these trials patients had to have a primary diagnosis of major depressive disorder (MDD) according to the DSM-IV or DSM-5 with a 17-item Hamilton Depression Rating Scale (HAM-D-17) score of 18 or higher. Additionally, patients had to be treatment-resistant, which included resistance (or intolerance) to several different antidepressants (e.g. SSRIs, SNRIs, tricyclic antidepressants (TCA), lithium augmentation, monoamine oxidase inhibitors) and electroconvulsive therapy (ECT). Patients were excluded if they had a primary or comorbid diagnosis of specific psychiatric or neurological disorders such as (but not limited to) bipolar disorder, schizophrenia, Parkinson's disease, dementia, and epilepsy. A complete list of the inclusion and exclusion criteria of these trials can be found in the Dutch National Trial Register (https://onderzoekmetmensen.nl/) and previously published manuscripts (see above).

### Surgery and treatment

Patients were bilaterally implanted with small DBS leads (1.5 ​mm contacts, 0.5 ​mm interspaces) in the vALIC (Supplementary Fig. 1) or slMFB target (Supplementary Fig. 2), which were then connected to a neurostimulator (vALIC: four-contact leads: 3389; stimulator: Activa PC or RC, Medtronic, Minneapolis, MN, USA; slMFB: eight-contact directional leads: DB-2202-30; stimulator: Vercise Gevia, Boston Scientific, Marlborough, MA, USA). Following a three-week recovery period, DBS parameter optimization was started. Parameter optimization consisted of a clinically guided systematic exploration of different parameter settings (e.g. different contacts, amplitudes, pulse widths) in order to find the optimal antidepressive effects with the least amount of adverse side effects.

### Study design and outcome measures

To assess the effect of DBS on different symptom dimensions, we retrospectively collected all available HAM-D-17 scores from the medical charts of the included patients. These included assessments during study visits from each trial as well as assessments from clinical follow-up visits before, during and after the trials. Since both trials included a blind, crossover phase in which the stimulation was turned off at some point in that phase, all measurements between the start and end of the crossover phase were excluded. The HAM-D-17 is a 17-item questionnaire which measures the severity of depressive symptoms. Higher scores indicate more severe depressive symptoms with a maximum score of 52. As an adjunct to the HAM-D-17 we additionally collected MADRS scores. The MADRS is a 10-item questionnaire used to measure depressive symptom severity. Higher scores indicate more severe depressive symptoms with a maximum score of 60.

The symptom dimensions of the HAM-D were defined based on an exploratory factor analysis by Wade et al., (2020) [8], because this was performed on a mostly treatment-resistant sample of patients with MDD (n ​= ​111), which best resembled our cohort. The exploratory factor analysis resulted in a 3-factor solution. Factor 1 consists of the items measuring work and interests, weight loss, psychomotor retardation, and depressed mood. Factor 2 consists of the items measuring somatic gastrointestinal symptoms, hypochondriasis, feelings of guilt, genital symptoms, general somatic symptoms, somatic anxiety, anxiety psychic, and psychomotor agitation. Factor 3 consists of the items measuring early insomnia, middle insomnia, and late insomnia. From this point onwards we will refer to these factors as the affective/anhedonia dimension, the somatic/anxiety dimension, and the insomnia dimension respectively. The suicide and insight items did not adequately load on any of these factors and were therefore not included in the analysis.

The symptom dimensions of the MADRS were defined based on an exploratory factor analysis by Borentain et al., (2022) [7], because this was performed on a sample of patients with TRD (identified in 223 patients and verified in 342 patients), which best resembled our cohort. The exploratory factor analysis resulted in a 3-factor solution. Factor 1 consists of the items apparent sadness, reported sadness, lassitude, and inability to feel. Factor 2 consists of the items inner tension, reduced sleep, reduced appetite, and concentration difficulties. Factor 3 consists of the items pessimistic thoughts and suicidality. From this point onwards we will refer to these factors as the affective/anhedonia dimension, the anxiety/vegetative dimension, and the hopelessness dimension respectively.

### Statistical analysis

#### Longitudinal analyses

All statistical analyses were performed in R version 4.2.1 (R Core Team, 2022). In order to assess the trajectory of the symptom changes in the different dimensions during a DBS response, we initially only included patients in the analyses who showed an overall symptom reduction of at least 25 ​% (considered partial response in DBS literature) at any time after stimulation onset, which we considered a clinically relevant reduction in symptoms. To assess symptom dimension trajectories starting from the point of (partial) response onset, we started with determining the timepoint of the first assessment which showed an overall symptom reduction of at least 25 ​% compared to the postoperative baseline assessment (day 0; DBS off), and assigned it as day 1. Note that the duration of time between what is defined as day 0 and day 1 therefore varies. The timepoints of all the subsequent assessments after this initial response were adjusted to be relative to day 1. As each symptom dimension contained different numbers of items they each had different maximum scores. Therefore, in order to be able to accurately compare the symptom dimension scores we normalized the scores on each of the symptom dimensions by dividing it by the maximum score on that dimension (HAM-D-17: affective and anhedonia: 14, somatic/anxiety: 26, and insomnia: 6; MADRS: affective/anhedonia: 24, anxiety/vegetative: 24, and hopelessness: 12). The normalized scores therefore reflect the fraction of the maximum score within a particular dimension. Then, we analyzed the HAM-D-17 and MADRS data separately with two linear mixed models. Patients who did not reach a 25 ​% in symptom reduction at any time were excluded from these analyses. For each of the models, normalized dimension score was included as the dependent variable, and the interaction between dimension (levels for HAM-D-17: affective/anhedonia, somatic/anxiety, insomnia; MADRS: affective/anhedonia, anxiety/vegetative, hopelessness) and time period (levels: postoperative baseline, 0–1 months, 1–3 month, 3–6 months, 6–12 months, 1–2 years, 2–15 years) was included as the independent variable. Furthermore, days since postoperative baseline was included as an independent covariate after log-transformation with the natural log (ln (1+#days)) to meet linearity assumptions. A random intercept and the log-transformed number of days since postoperative baseline were included as a random slope with individual patients as the grouping variable in both models. As a post-hoc check we analyzed the data again with DBS target (vALIC/slMFB) added as a covariate in each model to take into account the potential differences in results between the two targets. *P*-values of ≤0.05 were considered statistically significant.

Finally, we performed sensitivity analyses to determine whether our results would be different if we only including patients in our models who showed an overall symptom reduction of at least 50 ​% (full response) at any time after stimulation onset, and assessed symptom dimension trajectories after the onset of this first full response.

#### Associations with response

To assess whether baseline symptom severity within the different symptom dimensions is associated with DBS response, we performed separate linear regression analyses for the HAM-D-17 and MADRS scales. We included patients irrespective of whether or not they achieved a (partial) response. In each of the models overall reduction in symptom severity was the dependent variable. We calculated the total symptom reduction (1-(score at follow-up/score at pre-operative baseline)) at the assessment closest to 365 days after the postoperative baseline measurement, as generally patients have reached a stable response (if any) by then (1 ​= ​100 ​% reduction, 0 ​= ​no change, −1 ​= ​100 ​% increase). However, at the time of the analyses the data collection of the slMFB trial was still ongoing. Therefore, some included patients did not yet have an assessment close to 365 days after the postoperative baseline. However, these patients may have reached a stable response after 13 weeks at the earliest (according to the slMFB trial protocol). Accordingly, patients were excluded from the analyses if their closest assessment to 365 days was less than 13 weeks after stimulation onset. First, to assess whether overall symptom severity at baseline is associated with DBS response, we added the total HAM-D-17 score/total MADRS score at baseline as the independent variable to the model. Additionally, we added gender, age at surgery, and the number of days since the postoperative baseline assessment after log-transformation with the natural log (ln (1+#days)) to the model as covariates, to take into account their potential associations with DBS response. Second, to assess whether the severity of symptoms in the different dimensions at baseline contributed more or less to a potential association between baseline symptom severity and DBS response, we additionally created a similar model but instead of the total HAM-D-17 and MADRS scores we added each of three dimension scores (HAM-D-17: affective/anhedonia, somatic/anxiety, and insomnia; MADRS: affective/anhedonia, anxiety/vegetative, and hopelessness) as separate independent variables to the model. The same covariates were added. As a post-hoc check we analyzed the data again with DBS target (vALIC/slMFB) added as a covariate in each model to take into account the potential differences in results between the two targets. *P*-values of ≤0.05 were considered statistically significant.

## Results

A total of 42 patients met the inclusion criteria of this retrospective chart review (vALIC/slMFB ​= ​25/17; M/F ​= ​13/29; age at surgery ​= ​50.24 ​± ​11.64 (mean ​± ​SD)). These patients received DBS surgery between March 2010 and May 2023 and their data was collected from March 2010 until August 2023. A summary of DBS parameter settings at last follow-up are provided in Supplementary Table 1 (vALIC) and Supplementary Table 2 (slMFB). No follow-up data with individual HAM-D-17 item scores were available from three patients with vALIC DBS. The average last HAM-D-17 follow-up of the remaining 22 vALIC patients was at 62.4 ​± ​47.7 months (mean ​± ​SD). The average HAM-D-17 last follow-up of the 17 slMFB patients was at 11.3 ​± ​7.3 months (mean ​± ​SD). No follow-up data with individual MADRS item scores were available from four patients with vALIC DBS. The average last MADRS follow-up of the remaining 21 vALIC patients was at 37.5 ​± ​34.7 months (mean ​± ​SD). The average MADRS last follow-up of the 17 slMFB patients was at 10.6 ​± ​7.7 months (mean ​± ​SD).

### Longitudinal analyses

Supplementary Table 3 provides the number of unique patients and number of assessments within each time period within of the longitudinal analyses. The average number of days since postoperative baseline per time period is also provided.

#### HAM-D-17

**Fig. 1:**
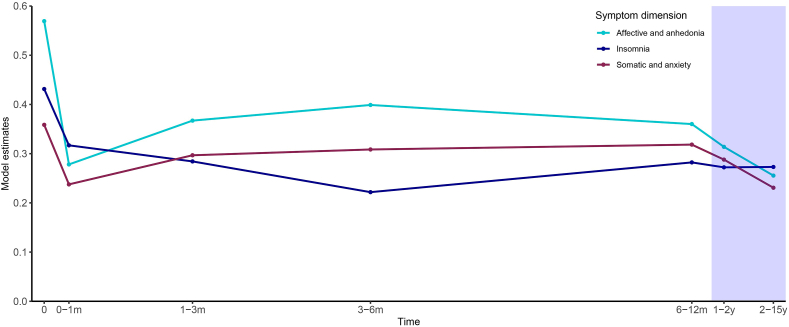
**Estimated trajectory of HAM-D-17 scores in different symptom dimensions after (partial) DBS response onset.** Displayed are the linear mixed model estimates, which reflect the estimated fraction of the HAM-D-17 total dimension scores (affective/anhedonia, somatic/anxiety, insomnia) present at different times after (partial) DBS response onset (0 ​= ​postoperative baseline). The model estimates are displayed at the mean number of days since the (partial) response onset of the assessments during each time period. For illustration purposes, after 6–12 months the x-axis is not shown according to scale (shaded purple). There was a significant interaction between HAM-D-17 dimension and time period (F (12,1655.46) ​= ​5.46, p ​< ​0.001). The (initial) reduction in symptom severity after (partial) response onset was larger in the affective/anhedonia dimension compared to the somatic/anxiety dimension and the insomnia dimension, and the insomnia dimension demonstrated a slower, more prolonged reduction.

To assess the HAM-D-17 symptom trajectories after the onset of an initial partial DBS response, we only included patients in the analyses who showed an overall symptom reduction of at least 25 ​% at any time after stimulation onset. Eight patients either did not have any HAM-D-17 assessments after stimulation onset (n ​= ​3), or never achieved a partial response based on HAM-D-17 scores during their treatment with DBS (n ​= ​5). The remaining 34 patients were included in the analysis (vALIC/slMFB ​= ​18/16; M/F ​= ​11/23; age at surgery ​= ​49.06 ​± ​11.90 (mean ​± ​SD)). The first (partial) response occurred at a median (Q1-Q3) of 71.5 (14.50–168.75) days after postoperative baseline. Afterward, symptom severity for each separate HAM-D-17 symptom dimension (affective/anhedonia, somatic/anxiety, and insomnia) was significantly lower during each time period compared to postoperative baseline (all p ​< ​0.05). Furthermore, there was a significant interaction between HAM-D-17 dimension and time period (F (12,1655.46) ​= ​5.46, p ​< ​0.001), indicating differences in symptom trajectories between dimensions. These effects remained significant after DBS target (vALIC/slMFB) was added as a covariate to the model and the effect of the covariate itself was non-significant. After inspection of the estimated model coefficients (illustrated in Fig. 1) an overall initial steep drop in symptom severity was observed in all dimensions within 0–1 month after the onset of the first (partial) response, followed by a period of about a year where symptom severity increased slightly while on the long-term (1–15 years) it decreased again. However, mainly two large differences between the symptom dimensions were noticeable: 1) the (initial) reduction in symptom severity was larger in the affective/anhedonia dimension compared to the somatic/anxiety dimension and the insomnia dimension, and 2) while symptom severity in the affective/anhedonia dimension and the somatic/anxiety dimension showed a similar trajectory, with an initial large reduction within 0–1 month after the onset of the first (partial) response, and the greatest reduction at 2–15 years, the insomnia dimension demonstrated a slower, more prolonged reduction, peaking at 3–6 months.

For the sensitivity analysis 28 patients were included (vALIC/slMFB ​= ​14/14; M/F ​= ​9/19; age at surgery ​= ​50.00 ​± ​10.99 (mean ​± ​SD)), who showed an overall symptom reduction of at least 50 ​% (full response) at any time after stimulation onset. The first full response occurred at a median (Q1-Q3) of 102 (23–418.75) days after postoperative baseline. This analysis showed that the interaction between HAM-D-17 dimension and time period remained significant (F (12,1002.67) ​= ​2.11, p ​= ​0.014), with and without the DBS target covariate. Supplementary Fig. 3 shows a very similar response trajectory as compared to the partial response analysis, especially for the affective/anhedonia dimension and somatic/anxiety dimension. However, the slower and more prolonged reduction in symptom severity in the insomnia dimension was no longer present. Instead, it showed an initial steep drop in symptom severity after response onset like the other two dimensions, followed by a relatively constant symptom severity for the first year, and a slight reduction and increase in symptom severity at 1–2 years and 2–15 years respectively.

#### MADRS

**Fig. 2:**
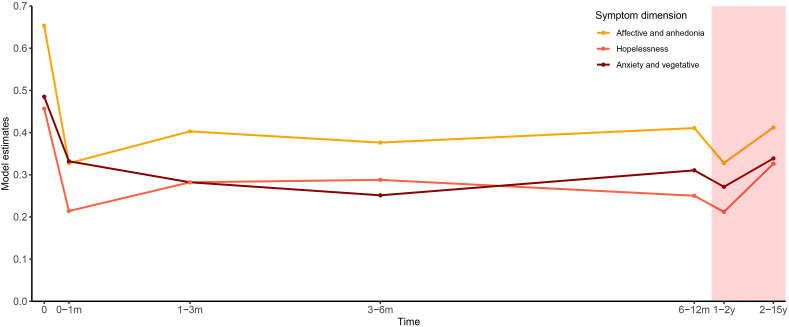
**Estimated trajectory of MADRS scores in different symptom dimensions after (partial) DBS response onset.** Displayed are the linear mixed model estimates which reflect the estimated fraction of the MADRS total dimension scores (affective/anhedonia, anxiety/vegetative, hopelessness) present at different times after (partial) DBS response (0 ​= ​postoperative baseline). The model estimates are displayed at the mean number of days since postoperative baseline of the assessments during each time period. For illustration purposes, after 6–12 months the x-axis is not shown according to scale (shaded red). There was a significant interaction between MADRS dimension and time period (F (12,938.73) ​= ​2.40, p ​< ​0.01). The (initial) reduction in symptom severity after (partial) response onset was larger in the affective/anhedonia dimension and the hopelessness dimension compared to the anxiety/vegetative dimension, and the anxiety/vegetative dimension demonstrated a slower, more prolonged reduction.

To assess the MADRS symptom trajectories after the onset of an initial partial DBS response, again, we only included patients in the analyses who showed an overall symptom reduction of at least 25 ​% at any time after stimulation onset. According to their charts, 17 patients either did not have any MADRS assessments after stimulation onset (n ​= ​4), or never achieved a partial response based on their MADRS scores during their treatment with DBS (n ​= ​13). The remaining 25 patients were included in the analysis (vALIC/slMFB ​= ​16/9; M/F ​= ​10/15; age at surgery ​= ​48.32 ​± ​10.96 (mean ​± ​SD)). The first (partial) response occurred at a median (Q1-Q3) of 84 (16–232) days after postoperative baseline. Afterward, symptom severity for each separate MADRS symptom dimension (affective/anhedonia, anxiety/vegetative, and hopelessness) was significantly lower during each time period compared to postoperative baseline (all p ​< ​0.001). Similar to the HAM-D-17 results, there was a significant interaction between MADRS dimension and time period (F (12,938.73) ​= ​2.40, p ​< ​0.01), indicating differences in symptom trajectories between dimensions. These effects remained significant after DBS target (vALIC/slMFB) was added as a covariate to the model and the effect of the covariate itself was non-significant. After inspection of the estimated model coefficients (illustrated in Fig. 2) an overall initial steep drop in symptom severity was observed in all dimensions within 0–1 month after the onset of the first (partial) response, followed by a period of about a year where symptom severity increased slightly and then remained relatively stable, while at 1–2 years it decreased slightly again, followed by a small increase at 2–15 years. Two large differences between the symptom dimensions were noticeable: 1) the (initial) reduction in symptom severity was larger in the affective/anhedonia dimension and hopelessness dimension compared to the anxiety/vegetative dimension, 2) while symptom severity in the affective/anhedonia dimension and the hopelessness dimension showed a similar trajectory, with an initial large reduction within 0–1 month after the onset of the first (partial) response and the greatest reductions at 0–1 month and 1–2 years, the anxiety/vegetative dimension demonstrated a slower, more prolonged reduction, peaking at 3–6 months.

For the sensitivity analysis on full responders (≥50 ​% decrease in MADRS) 21 patients were included (vALIC/slMFB ​= ​13/8; M/F ​= ​8/13; age at surgery ​= ​48.95 ​± ​11.38 (mean ​± ​SD)). The first full response occurred at a median (Q1-Q3) of 119 (15–397) days after postoperative baseline. This analysis showed that the interaction between MADRS dimension and time period was no longer significant (F (12,559.77) ​= ​0.9861, p ​= ​0.46), with and without the DBS target covariate. This suggests that in full responders the trajectory of the symptom severity in the different MADRS dimensions after response onset was similar (see Supplementary Fig. 4).

### Response associations

#### HAM-D-17

A total of 39 patients had HAM-D-17 assessments after at least 13 weeks of stimulation and were included in the linear regression analyses (vALIC/slMFB ​= ​22/17; M/F ​= ​11/28; age at surgery ​= ​49.41 ​± ​11.64 (mean ​± ​SD)). The other three patients were excluded as they did not have HAM-D-17 assessments after stimulation onset. As expected, the analyses showed that total HAM-D-17 score at baseline was significantly correlated with DBS response at the timepoint closest to one year (F (1,34) ​= ​11.91, p ​< ​0.01). Higher scores at pre-operative baseline were associated with a larger percentage symptom reduction. Our second model showed that only symptom severity in the somatic/anxiety dimension was significantly correlated with DBS response (F (1,32) ​= ​12.371, p ​< ​0.01), with higher scores at pre-operative baseline being associated with a larger percentage reduction in overall symptoms. Symptom severity at pre-operative baseline in the affective/anhedonia dimension tended to correlate with DBS response although this did not reach statistical significance (F (1,32) ​= ​3.80, p ​= ​0.06), and symptom severity at pre-operative baseline in the insomnia dimension was not significantly correlated with DBS response (F (1,32) ​= ​0.006, p ​= ​0.94). The statistical significance of these effects was not dependent on whether or not DBS target (vALIC/slMFB) was added as a covariate to the models and the effects of the covariate itself were non-significant.

#### MADRS

A total of six patients were excluded from the linear regression analysis with MADRS scores because they did not have MADRS assessments after stimulation onset (n ​= ​4) or did not have MADRS assessments after 13 weeks of stimulation (n ​= ​2). The linear regression analysis therefore included 36 patients (vALIC/slMFB ​= ​21/15; M/F ​= ​11/25; age at surgery ​= ​48.94 ​± ​11.83 (mean ​± ​SD)). We intended to include gender as a covariate in the analysis to take into account its association with DBS response, however, based on visual inspection of the data and normality tests, the assumption of normality was potentially violated for this model. When excluding gender from the analysis the assumption of normality was likely met. Regardless of the inclusion of gender in the model, neither the total MADRS score nor any of the dimension scores (affective/anhedonia, anxiety/vegetative, hopelessness) at pre-operative baseline were significantly associated with DBS response (all p ​> ​0.05).

The statistical significance of these effects was not dependent on whether or not DBS target (vALIC/slMFB) was added as a covariate to the models and the effects of the covariate itself were non-significant.

## Discussion

This retrospective chart study explored the trajectory of depressive symptom severity in different symptom dimensions after (partial) DBS response onset. Our main findings show that the first (partial) response occurred at a median of approximately 2.5 months after stimulation onset, and that each of the assessed symptom dimensions was significantly reduced throughout the recorded treatment course (last follow-up: 2–15 years) after (partial) DBS response onset. This suggests that symptom severity reductions are present and long lasting across all symptom dimensions after (partial) response onset. Additionally, we found a significant interaction between symptom dimension and time period based on both the HAM-D-17 and MADRS scores. After (partial) response onset, trajectories across dimensions were generally similar, although symptom severity reduced at a slower rate in the insomnia dimension (HAM-D-17) and the anxiety/vegetative dimension (MADRS) compared to the other dimensions of the respective scales. Our sensitivity analyses showed that when only full responders were included in these analyses, the slower responses on these specific symptom domains were no longer present. Additionally, as expected, we found that higher total HAM-D scores at baseline were associated with a larger percentage symptom reduction after DBS at the timepoint closest to a year. Further analysis showed that this association was specific to higher scores on the somatic/anxiety dimension. In contrast, we found no significant associations between DBS response and total MADRS scores or any of the MADRS symptom dimension scores at baseline.

Our findings are generally in line with the only study thus far assessing symptom reduction in different symptom dimensions after DBS for TRD [6]. This study showed that SCG DBS was associated with significant reductions in depressive symptoms in each assessed cluster of HAM-D-17 items (mood, anxiety, sleep, somatic) up to 12 months. However, it showed faster maximal improvements in mood symptoms compared to anxiety, sleep and somatic symptoms while we only observed this slower response for insomnia symptoms. This discrepancy may be due to differences in DBS target and/or analytical approaches. The SCG study examined changes in various symptom dimensions from the onset of stimulation, whereas our study focused on symptom severity changes after patients achieved at least a 25 ​% reduction in depressive symptoms. Due to differences in analytical approaches it is similarly difficult to compare our results to findings of similar research about other antidepressive therapies. However, comparable to our results, the factor analytic study on which we based our symptom dimensions showed that the affective/anhedonia dimension reduced a higher rate compared to the somatic/anxiety dimension and insomnia dimension after electroconvulsive therapy [8]. Additionally, antidepressive medications also affect insomnia symptoms to a lesser extent compared to core emotional symptoms (such as depressed mood and loss of interest) [5]. This may be because anxiety, insomnia, and somatic complaints are more secondary features of depression. The most common DBS targets are part of brain circuits implicated in core depressive symptoms [2], which could explain the more rapid and more pronounced effects on these symptoms. However, the findings from both ECT and pharmacological treatments suggest that, even without directly targeting these specific brain circuits, core depressive symptoms tend to show greater improvement than secondary symptoms.

In addition to the slower improvement in insomnia symptoms (HAM-D-17), we showed that anxiety and vegetative symptoms (MADRS) also improved at a slower rate after the onset of a (partial) DBS response compared to the other dimensions. Given that the HAM-D-17 items of the somatic/anxiety symptom dimension, which are also represented in the MADRS items of the anxiety/vegetative symptom dimension, did not show this slower response, it is likely the slower response is specifically associated with the sleep related items in both scales. Interestingly, as our sensitivity analyses with only full responders did not show these slower improvements, it is possible that it is specifically the partial response causing this effect. After inspecting the data of just the partial responders, we did indeed see this slower improvement pattern on these symptom dimensions. In addition, partial responders seemed to have less insomnia symptoms at baseline compared to full responders. However, as there were very few partial responders (HAM-D17: n ​= ​6; MADRS: n ​= ​4) we refrained from doing formal statistical analyses. The slower improvement in insomnia symptoms may be due to the working mechanism of the DBS. It is possible that insomnia symptoms only indirectly improve as a result of the reduction in symptoms in other dimensions. Although the precise working mechanisms of DBS in MDD are unknown it is generally thought that by targeting hubs in the reward circuitry of the brain (such as the vALIC and the slMFB) we can directly influence reward circuitry related processes such as mood regulation and motivation (e.g. 2), the disruption of which is linked to depressive symptoms such as anhedonia and a depressed mood in MDD [[10], [11], [12]]. Research shows that mood has a significant effect on sleep quality [13,14]. Additionally, studies have shown that negative emotion, rumination and worry, which also play an important role in depressive disorders [15], are associated with disturbed sleep [16,17]. Therefore, improvements in depressive symptoms may improve sleep quality. Furthermore, research also shows that the relationship between mood and sleep quality is reciprocal [13], and that the effect of sleep quality on mood is even larger [14]. Thus, long-term improvements in sleep after DBS for depression as shown by our study may act as a protective factor for depression relapse. However, it cannot be ruled out that DBS may also directly improve insomnia symptoms through the influence on the reward circuitry, as it recently has been suggested that there may be a link between sleep disturbance and reward processing deficits as supported by preliminary evidence [18]. On the other hand, transient sleep disorders are also frequently reported as a side effect of vALIC DBS [19]. This may be another explanation for why insomnia symptoms are slower to resolve. Including sleep studies in future DBS research would further clarify the exact relationship between DBS and improvements in insomnia symptoms. Moreover, our findings show that insomnia symptoms in patients with MDD who are being treated with DBS may need specific and additional clinical attention.

As expected, we showed that higher total symptom severity (HAM-D-17) at baseline was associated with a larger percentage symptom reduction after DBS at the timepoint closest to a year. Change scores can inherently be negatively correlated with baseline scores due to the regression to the mean phenomenon (e.g. Refs. [20,21]). However, further analysis showed that this association was specific to higher scores on the somatic/anxiety dimension. Sub-scale analyses are not inherently subject to the regression to mean phenomenon. However, scores on the somatic/anxiety dimension were highly correlated with total HAM-D-17 scores. Therefore, we cannot rule out that this association could also be at least partly explained by a regression to the mean effect, and it would be presumptive to suggest that baseline somatic and anxiety symptoms can predict DBS response. Nonetheless, the anxious MDD subtype has previously been associated with worse outcomes after treatment with antidepressants compared to non-anxious MDD [[22], [23], [24], [25], [26]]. Interestingly, our findings at least show that this is not the case for DBS and suggests that it may be a particularly effective intervention for patients with depression with relatively more somatic and anxiety symptoms. This is not entirely surprising given the success in treating patients with obsessive-compulsive disorder with DBS of the same and similar brain targets ((v)ALIC, Nucleus Accumbens, Ventral Capsule/Ventral Striatum) [27], which significantly reduces their often considerable anxiety symptoms (e.g. 19). Of note, while side effects such as restlessness and agitation have been reported for DBS targeting these regions (e.g. 9), they are typically stimulation related and transient. In contrast, total symptom severity and vegetative and anxiety symptom severity as measured with the MADRS was not associated with response in our sample. However, a difference between the two scales is that the vegetative and anxiety dimension of the MADRS only includes one item related to anxiety, which measures inner tension and seems to capture only the agitation and tension component of anxiety. The HAM-D-17, on the other hand, includes items on psychic anxiety, somatic anxiety, hypochondriasis and psychomotor agitation. Therefore, compared to the HAM-D-17 the MADRS is likely not sensitive enough to anxiety symptoms to be able to detect a relationship between the severity of anxiety symptoms and DBS response. Of important note, our regression analysis is not suited for reliable individual predictions of response based on baseline anxiety symptoms. Therefore, these findings should not be seen as a reason to only select patients with anxious depression for DBS.

To increase our sample size and therefore the power of our analyses we combined the data from patients with two different DBS targets (vALIC/slMFB). These targets are similar in the sense that they are both involved in the same reward circuitry of the brain and there is overlap in the white matter bundles which are within the volume of the DBS activated tissue. However, the stimulation sites for these bundles differ significantly: slMFB target stimulation occurs near the ventral tegmental area (VTA) in the midbrain, while vALIC target stimulation occurs near the NAc (although the slMFB also traverses through the (v)ALIC). Therefore, we cannot rule out that the DBS working mechanism and therefore the symptom trajectories after (partial) DBS response onset are different for these targets. Our post-hoc analyses showed that including DBS target as a covariate did not change the results and the covariate itself had non-significant effects, which suggests that symptom trajectories are not different for these targets. However, our analytical approach was not suited to investigate differences in (partial) response onset times between the two targets, and we suggest that this is separately investigated using appropriate and more frequent symptom severity assessments. In addition, to completely rule out differences between targets they should be directly compared in future well-powered studies. Other DBS targets for depression (e.g. SCG) should be investigated as well.

This study is the first to investigate the trajectory of symptom severity in different dimensions specifically after (partial) DBS response onset in depression. By focusing on the response trajectory after at least an initial partial response has occurred, our results were not convoluted with data from non-responders and we largely eliminated DBS parameter optimization induced noise, ensuring a more precise estimation of the response trajectories. Additionally, by combining the retrospective chart data from patients which were originally included in two separate clinical trials investigating DBS for TRD we ended up with a relatively large sample size with extensive long-term follow-up data.

However, our study had several limitations as retrospective chart review studies inherently carry several potential biases and constraints. For example, the quality of the data is reliant on the accuracy and completeness of the documentation. In addition, it is difficult to take into account different confounding factors (for example: benzodiazepine use may influence sleep and anxiety symptoms) due to the limited information available from the charts. Especially, after the patients finished the controlled part of the clinical trials, confounding factors may have played increasingly larger roles. And although the long-term follow-up data from up to 15 years is a strength of this study, it is also likely that different clinicians performed the assessments which, despite training, may have introduced some noise into the data. Furthermore, due to the retrospective chart design of the study we had no control over the amount of assessments per patient. The linear mixed models we used deal well with missing data by using maximum likelihood estimation. However, it is likely that patients who responded less well to DBS had more assessments as they tend to seek and receive more care. One way we tried to correct for this was by performing our sensitivity analyses which only included full responders. Still, especially longer term model estimates are likely biased due to this difference in the number of assessments, although we expect that this resulted in more conservative model estimates, more likely leading to an underestimation of DBS response rather than an overestimation.

Another limitation of the study was the purely explorative nature of our analyses. While this was valuable for uncovering patterns in the depressive symptom trajectories after a DBS response, our findings are preliminary and should be confirmed by controlled, prospective, hypothesis driven studies. We suggest that future studies specifically focus on the delayed improvement in insomnia symptoms after DBS intervention and the role of baseline anxiety symptoms in DBS outcome.

In conclusion, our findings demonstrate that DBS for TRD effectively treats all depressive symptoms long-term (up to 15 years) after (partial) response onset, although insomnia symptoms may be slower to resolve compared to other symptoms. Furthermore, our results suggest that patients with higher rates of somatic and anxiety symptoms may particularly benefit from DBS. These findings can provide prospective patients with more specific (but tentative) information on DBS effectiveness. Although our findings should be confirmed in more controlled prospective studies, these results are greatly encouraging and support the use of DBS in MDD patients with severe treatment resistance.

## Author contributions

Deep brain stimulation improves symptoms across all dimensions in treatment-resistant depression.

Runia, N., Mol, G. J. J., Denys, D. A. J. P. , Ardon, H., Beute, G., Bot, M., de Waardt, D. A., de Knijff, D. W. W., Mocking, R. J. T., Notten, P., Rutten, G. J. M., Schuurman, P. R., van den Munckhof, P., van Laarhoven, J., van Wingen, G. A. & Bergfeld, I. O.

Conceptualization: N. Runia, I.O. Bergfeld, G.A. van Wingen.

Methodology: N. Runia, I.O. Bergfeld, G.A. van Wingen.

Formal analysis: N. Runia.

Investigation: all authors.

Writing—original draft and visualization: N. Runia.

Supervision: D.A.J.P. Denys, I.O. Bergfeld, G.A. van Wingen.

Writing—review & editing: all authors.

## Declaration of competing interest

The authors declare the following financial interests/personal relationships which may be considered as potential competing interests: a part of the reported patients have been included in a previous investigator-initiated clinical trial funded by Medtronic Inc (25 DBS systems, in kind) and a grant from ZonMw (nr. 171201008). Nora Runia, Gosse Mol, Isidoor Bergfeld, Pepijn van den Munckhof, P. Richard Schuurman, Maarten Bot, Dieuwertje de Waardt, Dirk de Knijff, Hilko Ardon, Geert-Jan Rutten, Roel Mocking, Damiaan Denys, and Guido van Wingen currently execute an investigator-initiated clinical trial on deep brain stimulation for depression, in which another part of the reported patients is included. This trial is funded by Boston Scientific (24 DBS systems in kind) and a grant of ZonMw (nr. 636310016). P. Richard Schuurman acts as consultant for Boston Scientific and Medtronic on educational events. All other authors do not declare other conflicts of interest.
