# Supplementary material for: Deep brain stimulation improves symptoms across all dimensions in treatment-resistant depression
Source: Neurotherapeutics. 2025 Jun 10;22(5):e00623. doi: 10.1016/j.neurot.2025.e00623 (PMC12491800; doi:10.1016/j.neurot.2025.e00623)
Supplement: Multimedia component 1 [file mmc1.pdf]

**Supplementary Information for**

# Deep brain stimulation improves symptoms across all dimensions in treatment-resistant depression

N. Runia[a,b†], G.J.J. Mol[a], MD, prof.dr. D.A.J.P. Denys[a,b], MD, dr. H. Ardon[c], MD, G. Beute[c], MD, M. Bot[d], MD, D.A. de Waardt[e], MD, D.W.W. de Knijff[e], MD, dr. R.J.T. Mocking[a], MD, P. Notten[e], MD, dr. G.J.M. Rutten[c], MD, prof.dr. P.R. Schuurman[d], MD, dr. P. van den Munckhof[d], MD, J. van Laarhoven[e], MD, prof.dr. G.A. van Wingen[a,b], dr. I.O. Bergfeld[a,b]

[a] Amsterdam UMC location University of Amsterdam, Department of Psychiatry, Meibergdreef 9, Amsterdam, The Netherlands

[b] Amsterdam Neuroscience, Amsterdam, The Netherlands

[c] Department of Neurosurgery, ETZ, location Elisabeth, Tilburg, The Netherlands

[d] Amsterdam UMC location University of Amsterdam, Department of Neurosurgery, Amsterdam, the Netherlands

[e] Department of Psychiatry, ETZ, location Elisabeth, Tilburg, The Netherlands

[†]Corresponding author at: Amsterdam UMC location University of Amsterdam, Department of Psychiatry, Meibergdreef 9, 1105 AZ, Amsterdam, the Netherlands

E-mail: n.runia@amsterdamumc.nl

Telephone: +31645255607

## Supplementary Figure 1

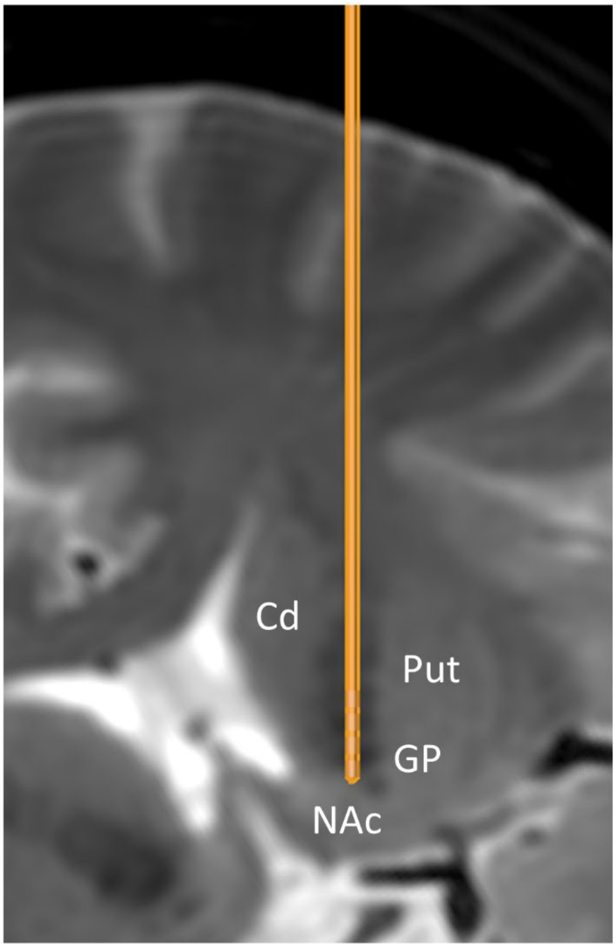


**Supplementary Figure 1. Anatomical planning of vALIC target.**

Inline coronal T2-weighted MRI planning of deep brain stimulation quadripolar electrode in the ventral part of the anterior limb of the internal capsule. *Abbreviations:* Cd, caudate nucleus; GP, globus pallidus; NAc, nucleus accumbens; Put, Putamen.

## Supplementary Figure 2

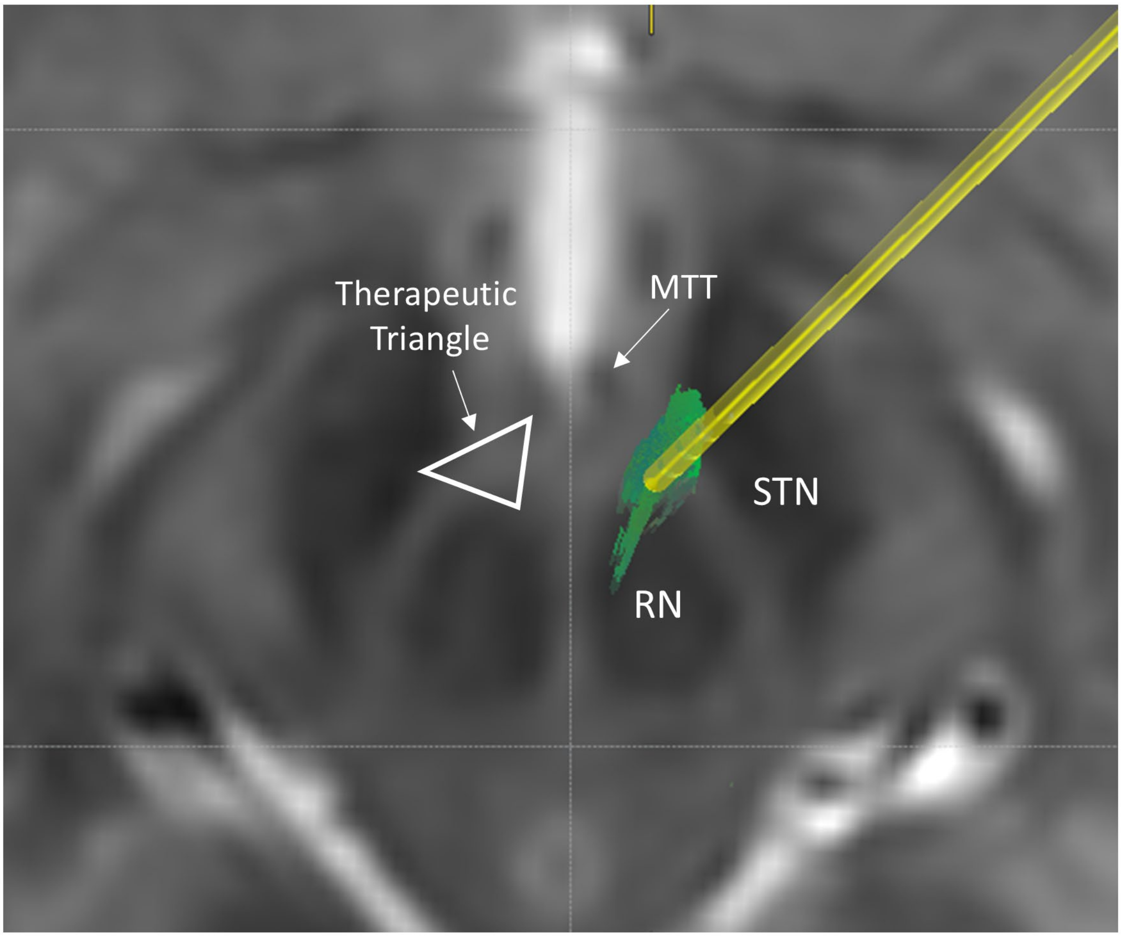


**Supplementary Figure 2. Tractographic planning of slMFB target.**

Axial T2-weighted MRI planning of deep brain stimulation quadripolar electrode in superolateral branch of the medial forebrain bundle (slMFB, displayed in green) at its location in the ventral tegmental area. At the axial T2 level with the largest diameter of the red nucleus (RN), the so-called 'therapeutic triangle' is displayed, which connects the mammillothalamic tract (MTT) with the anterior border of the RN and the medial border of the subthalamic nucleus (STN). This therapeutic triangle subsequently serves as a MR diffusion tensor imaging region of interest to depict the slMFB, similar to Coenen *et al.* (2017). *Abbreviations:* MTT, mammillothalamic tract; RN, red nucleus; STN, subthalamic nucleus.

## Supplementary Table 1

| Supplementary Table 1. vALIC DBS parameter settings at last follow-up (n=22) (Activa PC or RC, Medtronic) | | |
|---|---|---|
| **DBS parameter** | **Left electrode** | **Right electrode** |
| Electric potential, V (mean±SD) | 5.10±1.37 | 5.04±1.41 |
| Electric current, mA (mean±SD)* | 7.88±2.05 | 7.67±2.25 |
| Frequency | | |
| *130 Hz* | 12/22 | 12/22 |
| *140 Hz* | 1/22 | 1/22 |
| *180 Hz* | 9/22 | 9/22 |
| Pulse width | | |
| *60 µs* | 10/22 | 10/22 |
| *90 µs* | 8/22 | 8/22 |
| *120 µs* | 3/22 | 3/22 |
| *150 µs* | 1/22 | 1/22 |
| Monopolar (single contact) | 2/22 | 2/22 |
| Monopolar (multiple contacts) | 20/22 | 20/22 |
| Bipolar | 0/22 | 0/22 |

* For these devices the electric current is a non-adjustable parameter, resulting from the electric potential settings and the electrical resistance of the circuit.

## Supplementary Table 2

| Supplementary Table 2. slMFB DBS parameter settings at last follow-up (n=17) (Vercise Gevia, Boston Scientific) | | |
|---|---|---|
| **DBS parameter** | **Left electrode** | **Right electrode** |
| Electric current, mA (mean±SD) | 1.91±0.96* | 1.99±1.22 |
| Frequency | | |
| *130 Hz* | 16/16* | 17/17 |
| Pulse width | | |
| *60 µs* | 11/16* | 12/17 |
| *90 µs* | 5/16* | 5/17 |
| Monopolar (single contact) | 3/16* | 4/17 |
| Monopolar (multiple contacts) | 1/16* | 0/17 |
| Bipolar | 12/16* | 13/17 |

*The left electrode of one patient was turned off.

# Supplementary Table 3

**Supplementary Table 3. Specifics for the HAM-D-17 and MADRS longitudinal analyses**

| | Postoperative baseline | 0-1 m | 1-3 m | 3-6 m | 6-12 m | 1-2 y | 2-15 y |
|---|---|---|---|---|---|---|---|
| **≥25% response analyses** | | | | | | | |
| *HAM-D-17* | | | | | | | |
| n* | 34 | 34 | 28 | 23 | 18 | 22 | 10 |
| # assessments** | 34 | 73 | 79 | 93 | 106 | 103 | 95 |
| # days since postoperative baseline (mean±SD) | - | 10.0±10.4 | 60.1±16.3 | 132.0±24.4 | 262.0±53.3 | 503.0±103.3 | 2349.3±1232.4 |
| *MADRS* | | | | | | | |
| n* | 25 | 25 | 11 | 13 | 11 | 16 | 6 |
| # assessments** | 25 | 47 | 39 | 58 | 74 | 59 | 33 |
| # days since postoperative baseline (mean±SD) | - | 10.2±11.2 | 59.4±17.6 | 135.5±25.7 | 263.0±55.6 | 477.1±80.4 | 2665.8±1327.6 |
| **≥50% response analyses** | | | | | | | |
| *HAM-D-17* | | | | | | | |
| n* | 28 | 28 | 18 | 15 | 12 | 16 | 8 |
| # assessments** | 28 | 51 | 44 | 47 | 57 | 60 | 74 |
| # days since postoperative baseline (mean±SD) | - | 7.9±9.5 | 62.6±16.9 | 132.9±24.0 | 265.3±55.6 | 486.7±98.4 | 2603.4±1243.5 |
| *MADRS* | | | | | | | |
| n* | 21 | 21 | 5 | 8 | 6 | 13 | 4 |
| # assessments** | 21 | 30 | 15 | 27 | 45 | 40 | 31 |
| # days since postoperative baseline (mean±SD) | - | 6.8±9.9 | 56.5±17.9 | 137.7±26.8 | 265.7±51.8 | 475.0±75.1 | 2696.9±1272.3 |

* Number of subjects in the analysis within each time period. Assessments of missing subjects were estimated using maximum likelihood estimation.

** Number of assessments within each time period. Missing assessments were estimated using maximum likelihood estimation.

## Supplementary Figure 3

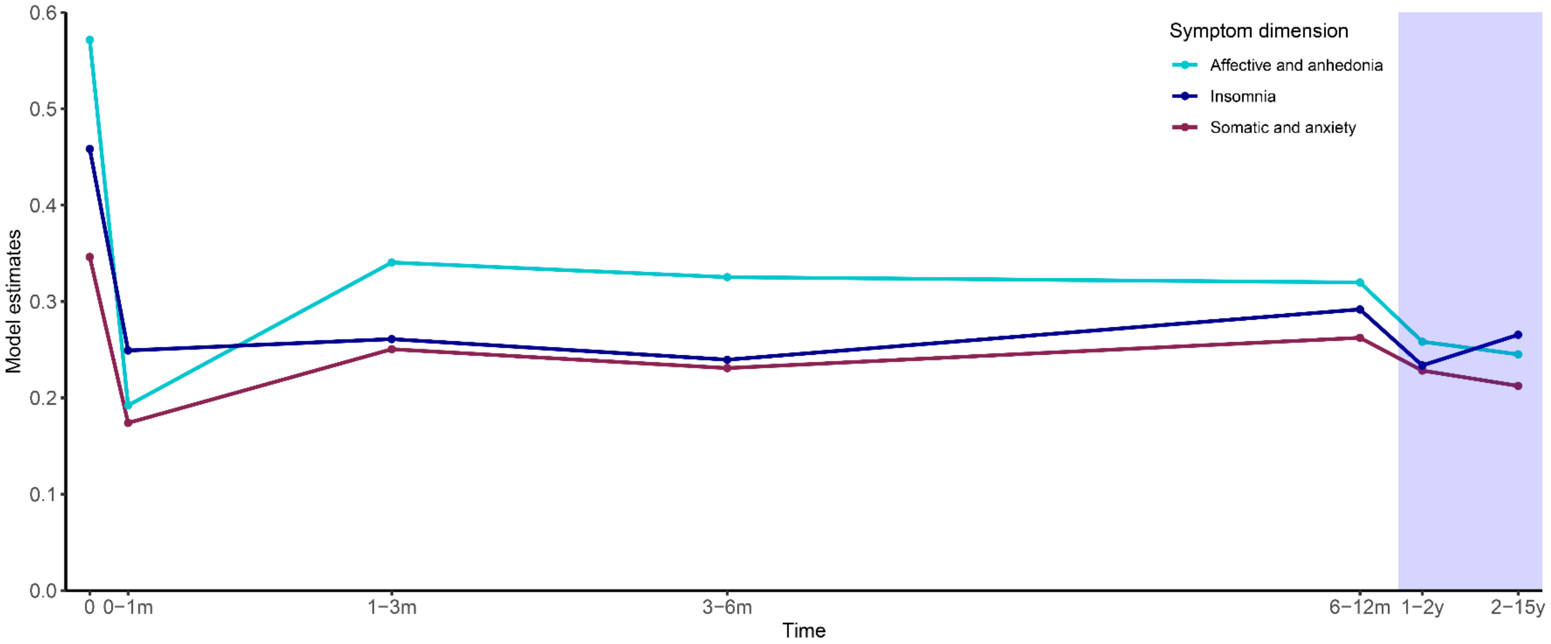


**Supplementary Figure 3. Estimated trajectory of HAM-D-17 scores in different symptom dimensions after full DBS response onset.** Displayed are the linear mixed model estimates, which reflect the estimated fraction of the HAM-D-17 total dimension scores (affective/anhedonia, somatic/anxiety, insomnia) present at different times after a full DBS response onset (0 = post-operative baseline). The model estimates are displayed at the mean number of days since the full response onset of the assessments during each time period. For illustration purposes, after 6-12 months the x-axis is not shown according to scale (shaded purple). There was a significant interaction between HAM-D-17 dimension and time period ($F(12,1002.67)=2.11$, $p=0.014$). The (initial) reduction in symptom severity after response onset was larger in the affective/anhedonia dimension compared to the somatic/anxiety dimension and the insomnia dimension. The slower and more prolonged reduction in symptom severity in the insomnia dimension observed in the initial analysis was no longer present.

## Supplementary Figure 4

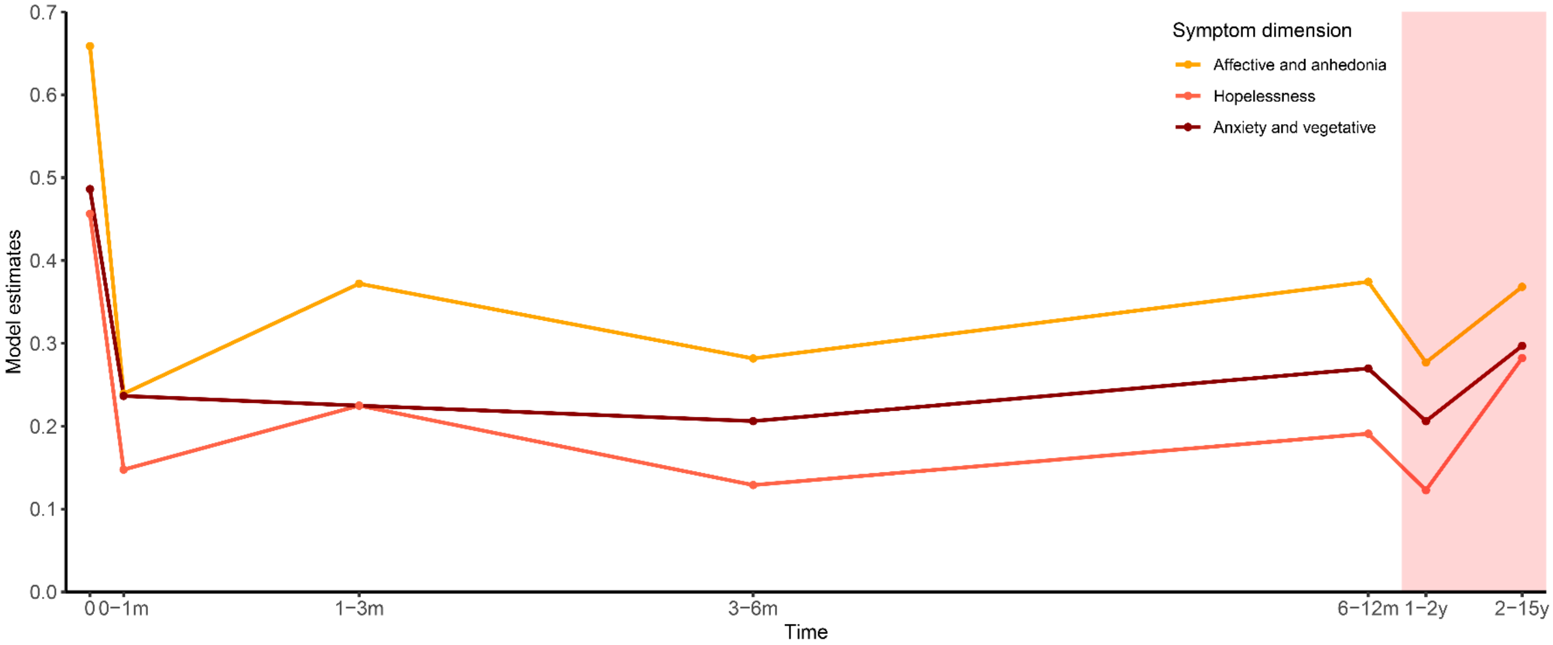


**Supplementary Figure 4. Estimated trajectory of MADRS scores in different symptom dimensions after full DBS response onset.** Displayed are the linear mixed model estimates, which reflect the estimated fraction of the MADRS total dimension scores (affective/anhedonia, anxiety/vegetative, hopelessness) present at different times after a full DBS response onset (0 = post-operative baseline). The model estimates are displayed at the mean number of days since the full response onset of the assessments during each time period. For illustration purposes, after 6-12 months the x-axis is not shown according to scale (shaded red). Each dimension shows a similar trajectory after response onset (dimension x time interaction effect: $F(12,559.77)=0.9861$, $p=0.46$).

## References

Coenen VA, Schlaepfer TE, Goll P, Reinacher PC, Voderholzer U, Van Elst LT, et al. The medial forebrain bundle as a target for deep brain stimulation for obsessive-compulsive disorder. CNS spectrums. 2017;22(3):282-9.
